# Inoculation of the *Leishmania infantum HSP70-II* Null Mutant Induces Long-Term Protection against *L. amazonensis* Infection in BALB/c Mice

**DOI:** 10.3390/microorganisms9020363

**Published:** 2021-02-12

**Authors:** Manuel Soto, Laura Ramírez, José Carlos Solana, Emma C. L. Cook, Elena Hernández-García, José María Requena, Salvador Iborra

**Affiliations:** 1 Centro de Biología Molecular Severo Ochoa (CSIC-UAM), Departamento de Biología Molecular, Universidad Autónoma de Madrid, 28049 Madrid, Spain; laura.ramirezg@gmail.com (L.R.); jc.solana@isciii.es (J.C.S.); jmrequena@cbm.csic.es (J.M.R.); 2 WHO Collaborating Centre for Leishmaniasis, National Centre for Microbiology, Instituto de Salud Carlos III, 28220 Madrid, Spain; 3 Department of Immunology, Ophthalmology and ENT, Complutense University School of Medicine and 12 de Octubre Health Research Institute (imas12), 28040 Madrid, Spain; emmaclare.cook@externo.cnic.es (E.C.L.C.); elena.hernandez@externo.cnic.es (E.H.-G.)

**Keywords:** *Leishmania amazonensis*, live vaccines, attenuated parasites, murine leishmaniasis, BALB/c mice, IFN-γ

## Abstract

*Leishmania amazonensis* parasites are etiological agents of cutaneous leishmaniasis in the New World. BALB/c mice are highly susceptible to *L. amazonensis* challenge due to their inability to mount parasite-dependent IFN-γ-mediated responses. Here, we analyzed the capacity of a single administration of the *LiΔHSP70-II* genetically-modified attenuated *L. infantum* line in preventing cutaneous leishmaniasis in mice challenged with *L. amazonensis* virulent parasites. In previous studies, this live attenuated vaccine has demonstrated to induce long-protection against murine leishmaniasis due to Old World *Leishmania* species. Vaccinated mice showed a reduction in the disease evolution due to *L. amazonensis* challenge, namely reduction in cutaneous lesions and parasite burdens. In contrast to control animals, after the challenge, protected mice showed anti-*Leishmania* IgG2a circulating antibodies accompanied to the induction of *Leishmania*-driven specific IFN-γ systemic response. An analysis performed in the lymph node draining the site of infection revealed an increase of the parasite-specific IFN-ϒ production by CD4^+^ and CD8^+^ T cells and a decrease in the secretion of IL-10 against leishmanial antigens. Since the immunity caused by the inoculation of this live vaccine generates protection against different forms of murine leishmaniasis, we postulate *LiΔHSP70-II* as a candidate for the development of human vaccines.

## 1. Introduction

*Leishmania amazonensis* parasites are one of the causative agents of different forms of American cutaneous leishmaniasis (ACL), including localized (LCL) and disseminated forms, as well as the most severe and incurable clinical form, anergic diffuse cutaneous leishmaniasis (DCL) [1]. The immunopathology of these forms varies from the existence of a predominant Th1 response against the parasite in LCL patients to the induction of Th2-mediated responses accompanied with a very limited production of IFN-ϒ against parasite antigens in patients with anergic DCL [1,2]. This species belongs to the *Leishmania* subgenus and coexists with different species of the *Viannia* subgenus such as *L. braziliensis*, *L. guyanensis* or *L. panamensis* [3]. Although murine models do not reproduce the complexity of the disease in humans, they have been used to advance the analysis of the immune response against *Leishmania.* In the most paradigmatic model of CL, the infection with the Old World cutaneotropic *L. major* species, effective immunity depends on the induction of a parasite-specific IFN-ϒ-mediated response. This response is able to induce nitric oxide-dependent killing of amastigotes in infected macrophages and is observed in a disease-resistant model where C57BL/6 mice are infected. On the other hand, susceptibility is associated to the induction of parasite-specific IL-4 and IL-10-mediated responses, as in the *L. major*-BALB/c model [4]. In the case of *L. amazonensis*, BALB/c mice fail to resolve the infection due to the difficulty in generating Th1 responses in addition to the parasite-specific production of IL-10 [5,6].

BALB/c mice have been extensively employed for the development of therapeutic strategies [7], as well as for the design of vaccines. Different strategies of vaccination have been tested in the *L. amazonensis*-BALB/c model using different sources of antigens, including the use of *L. amazonensis* total parasite extracts administered in different preparations [8,9,10,11,12], parasite protein fractions [13] or *L. amazonensis*-defined antigens, usually injected as DNA vaccines [14,15]. In addition, some protection has been reported for vaccines composed by protein extracts [16] or defined antigens [17,18] from other *Leishmania* species.

In recent years, the use of attenuated strains for the generation of vaccines against visceral leishmaniasis (VL) [19] or CL [20] has proven to be an interesting alternative to the immunization of subunit-based vaccines. In this sense, there are very few references regarding the analysis of these vaccines in murine models of infection by *L. amazonensis*. Recently, a single inoculation of photoinactivated *L. amazonensis* promastigotes was injected in the ear dermis of BALB/c mice and induced short-term protection against a virulent homologous challenge in the tail base [21]. In addition, BALB/c mice were injected with *L. infantum* promastigotes bearing genes encoding for toxic proteins able to kill the amastigote forms in the vertebrate host. They presented a less severe disease when challenged with *L. amanonensis*, namely a reduction in skin lesions and parasite load compared to the animals of a control group [22]. Regarding the use attenuated lines generated by genetic modification, it has been shown that BALB/c mice vaccinated with a dihydrofolate-reductase thymidylate synthase *L. major* null mutant (*Lmdhfr-ts*^–^) were partially protected against a *L. amazonensis* infective challenge administered in the short term [23].

Evidences on the use of live vaccines against CL due to *L. amazonensis* infections are still scarce. In this work, we present the effect of vaccination with the attenuated strain of *L. infantum LiΔHSP70-II* in the subsequent evolution of cutaneous leishmaniasis caused by an infection with *L. amazonensis*. Vaccination with this attenuated cell line was able to induce an immune response capable of controlling CL [24,25] and VL [26] development caused by *L. major* or *L. infantum* infectious challenge in BALB/c mice, respectively. We present here data regarding the evolution of the CL disease as well as the immune response elicited in vaccinated and unvaccinated mice after *L amazonensis* challenge.

## 2. Materials and Methods

### 2.1. Mice, Parasites, Vaccination, and Challenge

Female BALB/cOlaHsd mice (6 weeks old at the beginning of the assays) were purchased from Envigo (Huntingdon, UK). The procedures were achieved according to the Directive 2010/63/UE-Recommendation 2007/526/EC (European Union) and to the RD53/2103 from the Spanish Government. Experiments were approved by the Centro de Biología Molecular Severo Ochoa by the Animal Care and Use Committee (reference CEEA-CBMSO 23/243), the Bioethical Committee of the Spanish Consejo Superior de Investigaciones Científicas (reference 795/2019) and the Government of the Autonomous Community of Madrid (Spain) under the reference PROEX134/19.

The following *Leishmania* parasites species were employed: The attenuated cell line *L. infantum* MCAN/ES/96/BCN150 [*Δhsp70-II::NEO/Δhsp70-II::HYG]* [27] for vaccination, *L. infantum* (MCAN/ES/96/BCN150) for preparation of soluble leishmanial antigen (SLA) employed in the analysis of the anti-parasite humoral response post-vaccination (see below), and *L. amazonensis* (IFLA/BR/67/PH8), generously provided by Dr. Javier Moreno (ISCII, Spain) for challenge and SLA preparation (post-challenge humoral and cellular analyses).

Promastigote forms were grown in Schneider medium (Gibco, NY, USA) supplemented with 10% Fetal Calf Serum (FCS) (Sigma, MO, USA), 100 U/mL of penicillin and 100 µg/mL of streptomycin (complete Schneider medium) at 26 °C. Complete medium was additionally supplemented with 20 μg/mL of G418 (Sigma, MO, USA) and 50 μg/mL of hygromycin (Sigma, MO, USA) for growing the attenuated cell line.

### 2.2. Vaccination, Challenge, and Disease Follow-Up

Vaccination was performed subcutaneously (s.c.) as described in [26]. Briefly, 1 × 10^7^ *LiΔHSP70-II* promastigotes (in 30 µl of phosphate saline buffer (PBS)) were single-administered in the right footpad. In all cases, a control group was established (receiving only PBS). For challenge, BALB/c mice from the control or the vaccinated groups received 5 × 10^4^ (low dose group: n = 5 per group) or 5 × 10^6^ (high dose group: n = 8 per group) *L. amazonensis* stationary promastigotes s.c. (in 30 µL of PBS) in the left footpad. Challenge was done 12 weeks after vaccination. All the experiments were performed independently two times. Data shown in the figures represent one experiment with similar results obtained in a second one using the same number of animals. In all the assays, samples were processed and analyzed independently from each mouse.

Infection follow-up was performed by assessing footpad swelling with a metric digital caliper. Lesion size was expressed as thickness of the *L. amazonensis* left footpad minus thickness of the right footpad. For parasite burden quantification a limiting dilution assay was performed as described in [25,28]. Briefly, after sacrifice the popliteal lymph nodes draining (DLN) the site of vaccination (right popliteous) or challenge (left popliteous), the left footpad, the spleen and a piece of approximately 20 mg of the liver were independently homogenized and filtered through 70 µm cell strainers (Corning Gmbh, Kaiserslautern, Germany) to obtain cell suspensions. Individual samples were serially diluted (1/3) in triplicates in a 96-well flat-bottomed microtiter plate containing complete Schneider medium supplemented or not by 20 μg/mL G418 and 50 μg/mL hygromycin. The number of viable parasites was determined from the highest dilution at which promastigotes could be grown up to 14 days of incubation at 26 °C and is indicated per whole organ.

### 2.3. Sera Preparation and ELISA

Sera were obtained from blood samples taken 12 weeks after vaccination and 14 weeks after challenge (low dose) or 11 weeks after challenge (high dose). SLA were prepared by three freezing and thawing cycles of stationary promastigotes suspended in PBS followed by centrifugation for 15 min at 12,000× *g* using a microcentrifuge. For ELISA, *L. infantum* or *L. amazonensis* SLA was employed to analyze the reactivity of the samples before and after challenge, respectively. Assays were performed as previously described [25] using 96-well MaxiSorp plates (Nunc, Roskilde, Denmark) (1 ng per well). The sera end-point titer of IgG1 and IgG2a specific for SLA was calculated as the inverse value of the highest serum dilution factor giving an absorbance > 0.15. Anti-IgG1 or anti-IgG2a horseradish peroxidase-conjugated anti-mouse immunoglobulins were purchased from Nordic BioSite (Täby, Sweden). In all the assays, samples were collected and processed individually.

### 2.4. In Vitro Spleen Cell Stimulation and Analysis of Cytokine Concentration in Culture Supernatants

For the analysis of the systemic production of cytokine, primary cultures were established from the spleen as described above, but using RPMI complete medium (RPMI medium (Sigma) supplemented with 10% heat-inactivated FCS, 20 mM L-glutamine, 200 U/mL penicillin, 100 µg/mL streptomycin and 50 µg/mL gentamicin) instead of Schneider medium. Cells (5 × 10^6^ per ml) were cultured during 72 h at 37 °C in 5% CO_2_ in the absence or in the presence of *L. amazonensis* SLA at 12 µg/mL or Concanavalin A (ConA) at 1 µg/mL of final concentration. The levels of IFN-γ, IL-10 or IL-4 in culture supernatants were determined by sandwich ELISA using commercial kits (Thermo Fischer Scientific, Waltham, MA, USA). In all the assays, samples were collected and processed individually.

### 2.5. In Vitro DNLs Cell Stimulation and Analysis of Cytokine Concentration in Culture Supernatants

DLNs cell cultures (left popliteous) were established in RPMI complete medium. For anti-CD3/antiCD28 stimulation, 96-well High Bind COSTAR plates were coated for 2 h with at 37 °C with antiCD3ε (Clone 145-2C11; 1 μg per well). DLN cells (1 × 10^6^ per well) were cultured during 12 h at 37 °C in 5% CO_2_ in the presence of 0.2 µg of anti-CD28 (clone 37.51). Plates and antibodies were purchased by Thermo Fisher (Waltham, MA, USA). For control, cells were cultured in the same conditions in the absence of both antibodies.

For SLA stimulation, DLN cells (2 × 10^6^ cells/mL) were co-cultured during 48 h at 37 °C in 5% CO_2_ with granulocyte-macrophage colony-stimulating factor (GMCSF) bone marrow-derived cells (BM-DCs) stimulated or not with SLA (4 × 10 ^5^ cells/mL). BM-DCs cells were obtained from BM suspensions by culturing for 7 days in RPMI complete medium supplemented with 20 ng/mL of recombinant GMCSF (Peprotech, London, UK). For stimulation, BM-DCs were pulsed with *L. amazonensis* SLA (3 µg/mL) the last 24 h of culture. The levels of IFN-γ, IL-10 or IL-4 in culture supernatants were determined by sandwich ELISA using commercial kits (Thermo Fischer Scientific). In all the assays, samples were collected and processed individually.

### 2.6. Analysis of T Cell Populations by Flow Cytometry

For identification of T cell producing cytokines in *L. amazonensis* infected DLNs, cell cultures were established and co-cultured with BM-DCs pulsed or not with *L. amazonensis* SLA as described above, but during 24 h. In addition, cultures were treated with 10 µg/mL brefeldin A (Sigma) the last 6 h. Then, cells were harvested, washed in PBS with 1% heat-inactivated FCS (PBSw) and incubated with Mouse BD Fc Block (BD Biosciences; Franklin Lakes, NJ, USA). Next, cells were stained with antibodies specific for the CD3 (clone 145-2C11; APC), CD4 (clone RM4-5; BV570) and CD8 (clone 53-6.7; FITC) during 30 min at 4 °C. After washing in PBSw cells were fixed and permeabilized with Cytofix/Cytoperm (BD Biosciences, Franklin Lakes, NJ, USA) during 30 min at 4 °C. Next, PE/Cy7 anti-mouse IFN-γ (clone XMG1.2) antibody was added for 30 min at 4 °C. Finally, cells were washed and analyzed. Labelled antibodies were purchased from BioLegend (San Diego, CA, USA). Samples were analyzed using a FACS Canto II flow cytometer and FACSDiva Software (BD Biosciences, Franklin Lakes, NJ, USA) and processed and plotted with FlowJo Software (FlowJo LLC, Ashland, OR, USA). In all the assays, samples were collected and processed individually.

### 2.7. Statistical Analysis

Data (samples *n* ≥ 8) were analyzed by the D′Agostino and Pearson normality test. Parametric data were analyzed by a two-tailed Student t-test. Non-parametric data (or data with *n* < 8) were analyzed by a Mann–Whitney test. Differences were considered significant when *p* < 0.05. Statistical analysis was performed using the Graph-Pad Prism 5 program (GraphPad Software, San Diego, CA, USA).

## 3. Results

### 3.1. The LiΔHSP70-II Based Vaccine Induces Long-Term Protection Against a L. amazonensis Virulent Challenge in BALB/c Mice

**Figure 1:**
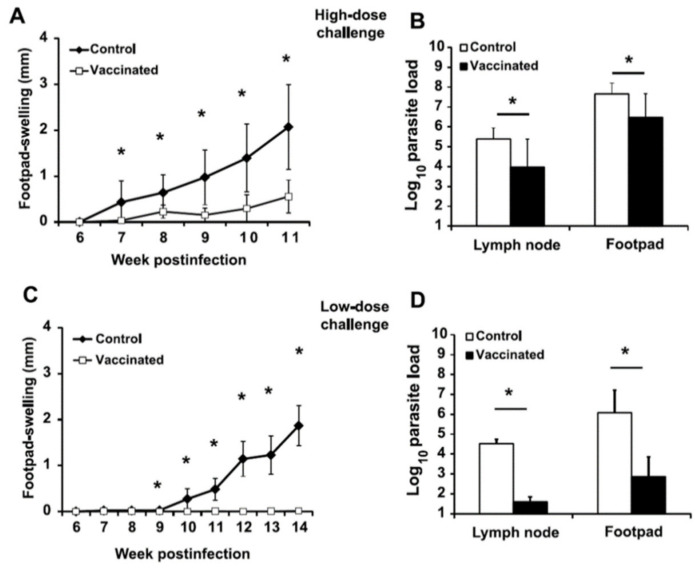
Course of *Leishmania amazonensis* infection in control and vaccinated mice. BALB/c mice were inoculated with PBS (control) or with 1 × 10^7^ *LiΔHSP70-II* attenuated promastigotes suspended in PBS (vaccinated) in the right footpad. At week 12 after vaccination control and vaccinated mice were challenged with 5 × 10^6^ ((**A**,**B**); high dose, *n* = 8) or 5 × 10^4^ ((**C**,**D**); low dose, *n* = 5) stationary *L. amazonensis* promastigotes in the left footpad. In (**A**) and (**C**), swelling was calculated weekly and it is given as the difference in thickness between the challenged and the contralateral footpad (mean ± standard deviation (SD)). Parasite burden was individually determined by a limiting dilution assay in the left footpad and the left popliteal lymph node at week 11 (**B**) or 14 (**D**) after infectious challenge and it is represented as the mean + SD. * (*p* < 0.05; Student′s t-test). Results from one out of two similar experiments are represented. No parasites were detected in the liver or the spleen of mice from control or vaccinated groups in any of the experiments.

We first analyzed the long-term effect of vaccination with the *LiΔHSP70-II* attenuated line in the development of murine leishmaniasis due to the infection with a high dose of *L. amazonensis* (5 × 10^6^ stationary phase promastigotes). For comparison, a group of mice inoculated with the vaccine diluent (PBS) at the time of vaccination was also challenged with the infective species. Skin lesions appeared from the seventh week in both groups of animals (Figure 1A). Footpad swelling decreased significantly in vaccinated animals compared to controls (Figure 1A) until week 11 after challenge.

At week 11 after challenge *L. amazonensis* promastigotes were detected in the left footpad and the corresponding draining lymph node (DLN; left popliteal) in control and vaccinated mice groups (Figure 1B). Remarkably, lower parasite burdens (1.68-log_10_ in the DLN, *p* = 0.0003; 1.51-log_10_ in the footpad, *p* = 0.002) were observed at both locations in the vaccinated animals with regard of the mice from the control group (Figure 1B). No *Leishmania* parasites (vaccine or infectious challenge) were found in internal organs such as liver and spleen (additional file 1: Figure S1). The persistent presence of the parasite composing the vaccine was only found in the right popliteal node draining the site of vaccination (additional file 1: Figure S1).

A complete control of the time-dependent development of pathological lesions due to vaccination was observed when animals were challenged with 5 × 10^4^ stationary *L. amazonensis* promastigotes (low dose; Figure 1C). In fact, in the fourteenth week post-challenge, all vaccinated animals presented an infected footpad similar to the uninfected one (Figure 1C). In spite of the lack of lesions, vaccinated animals had *L. amazonensis* parasites in the infected footpads and the corresponding DLNs. However, parasite burden was several orders of magnitude smaller in vaccinated mice compared to unvaccinated controls (2.92-log_10_ in the DLN, *p* = 0.0001; 3.22-log_10_ in the footpad, *p* = 0.005) (Figure 1D).

### 3.2. Vaccination with the LiΔHSP70-II Attenuated Cell Line Modulates the Profile of the Anti-Leishmanial Antibodies and Parasite-Specific Systemic Cytokine Secretion after Challenge with L. amazonensis

**Figure 2:**
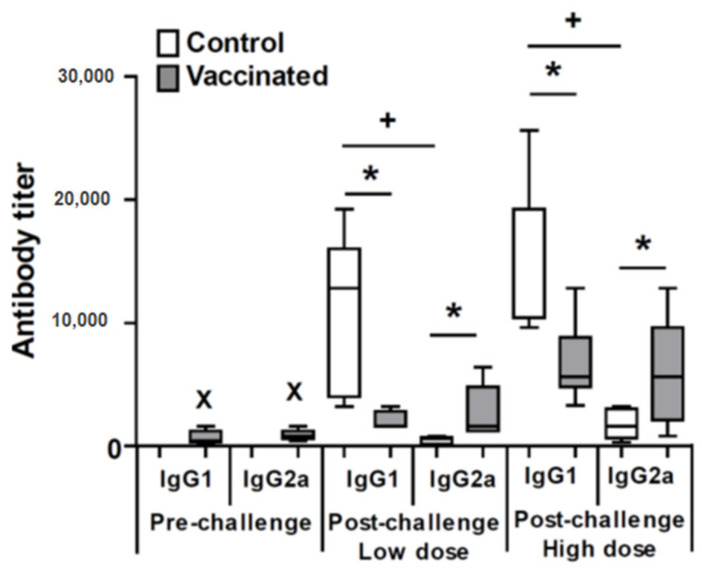
Humoral responses against *Leishmania* parasites. The reciprocal end-point titers of the IgG1 and IgG2a anti-leishmanial subclasses were analyzed by ELISA 12 weeks after the inoculation of the *LiΔHSP70-II* attenuated line (pre-challenge; *n* =13; *L. infantum* soluble leishmanial antigens (SLA)), 14 weeks after challenge (5 × 10^4^ promastigotes; low dose *n* = 5) and 11 weeks after challenge (5 × 10^6^ promastigotes; high dose *n* = 8) with *L. amazonensis* using SLA of this last species. Data are represented as whisker (min to max) plots and represent one out of two experiments with similar results. ^X^ (*p* < 0.05 statistical differences among titer from the pre-challenge and both post-challenge mice; * (*p* < 0.05 between titers from control and vaccinated groups); + (*p* < 0.05 between IgG1 and IgG2a form animals of the control group) analyzed by Kruskal-Wallis test.

Next, we determined the parasite-specific (anti-SLA) IgG1 and IgG2a antibody titer in the sera from animals from the low dose and high dose models. For comparison, we determined the humoral response against the parasite in all mice at the time of challenge (week 12 after vaccination). As expected, at that time only vaccinated mice showed anti-leishmanial antibodies of both, IgG1 and IgG2a subclasses, in similar levels to these previously described [25,26] (Figure 2). After infectious challenge at the time of the sacrifice, mice from both control groups showed an IgG1 predominant antibody response against the parasite antigens, being the titer of the anti-SLA IgG1 subclass significantly higher than the IgG2a subclass (*p* = 0.01, low dose; *p* = 0.0009, high dose) (Figure 2). On the contrary, vaccinated mice groups showed similar titers or the anti-SLA IgG1 and IgG2a antibodies against the parasite. Infection causes a boost in antibody production, since both subclasses titers were higher in magnitude after *L. amazonensis* infection compared to these observed pre-challenge: IgG1, *p* = 0.023, low dose; *p* = 0.0043, high dose; IgG2a, *p* = 0.049, low dose; *p* = 0.0176, high dose (Figure 2). Comparison of the anti-*Leishmania* antibody titer between animals of the control and vaccinated groups after challenge demonstrate that vaccination resulted in a significant increase in the production of antibodies of the IgG2a subclass against SLA (*p* = 0.012, low dose; *p* = 0.027, high dose) and a significant decrease (*p* = 0.015, low dose; *p* = 0.0029, high dose) in the titer of the anti-SLA IgG1 subclass after infection with *L. amazonensis* (Figure 2).

**Figure 3:**
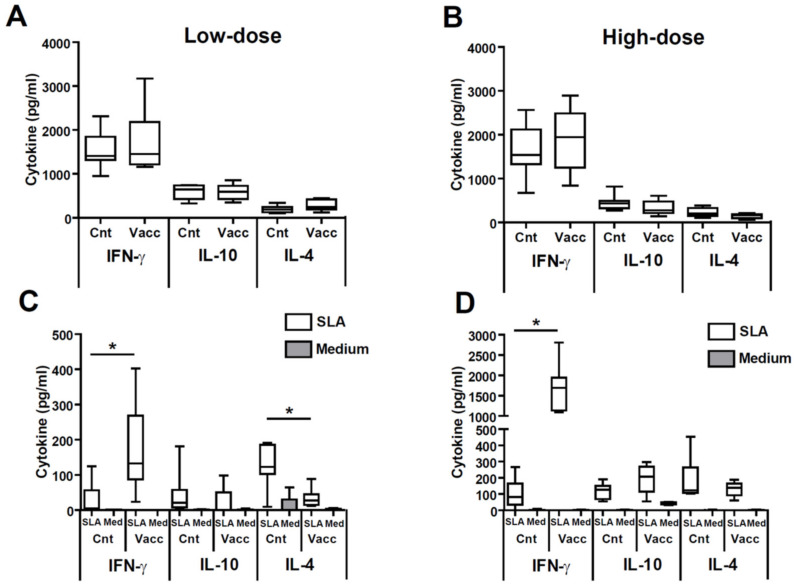
Analyses of the systemic production of cytokines. BALB/c mice were inoculated with PBS (control) or with 1 × 10^7^ *LiΔHSP70-II* attenuated promastigotes suspended in PBS (vaccinated) in the right footpad. At week 12 after vaccination control and vaccinated mice were challenged with 5 × 10^4^ ((**A**,**C**); low dose, *n* = 5) or 5 × 10^6^ ((**B**,**D**); high dose, *n* = 8) stationary *L. amazonensis* promastigotes in the left footpad. Animals were sacrificed at week 11 (high dose) or week 14 (low dose) post-challenge and their spleen cells were independently cultured in the presence of ConA (1 µg/mL; (**A**,**B**)), *L. amazonensis* SLA (12 µg/mL) or in medium alone (**C**,**D**). The levels of IFN-γ, IL-10, and IL-4 were assessed by ELISA in culture supernatants and are shown as whisker (min to max) plots. * (*p* < 0.05) shows the statistical differences between control and vaccinated groups (Mann–Whitney test). Results from one out of two similar experiments are represented.

Since the production of IgG1 or IgG2a has been related to the induction of Th2 or Th1 response, respectively [29], our data suggest that vaccination down-modulates the predominant *L. amazonensis*-mediated Th2 response generated in the control animals, increasing the production of a pro-inflammatory Th1-mediated response against the parasite. To test this hypothesis, we studied the systemic cytokine response elicited in animals infected with *L. amazonensis*. Polyclonal stimulation of splenocytes with the mitogen ConA generated similar levels of cytokines in mice from the control and vaccinated groups (Figure 3A,B). On the contrary, the secretion of SLA-specific cytokines (parasite stimulus) was different in control and vaccinated mice (Figure 3C,D). In high and low dose models, vaccinated mice produced significantly increased levels of IFN-γ with respect controls: *p* = 0.0008, low dose; *p* = 0.0002, high dose (Figure 3C,D). Similar levels of parasite-specific IL-10 were detected in supernatants established from control or vaccinated mice after SLA stimulation independent of the dose (Figure 3C,D). A decrease in the production of the Th2-related IL-4 cytokine upon stimulation with SLA was observed in mice from the vaccinated group that was only significant in the animals from the low dose model (*p* = 0.016) (Figure 3C).

### 3.3. Vaccinated Animals Show a Local Cytokine Response Directed towards the Production of IFN-γ against the Parasite

**Figure 4:**
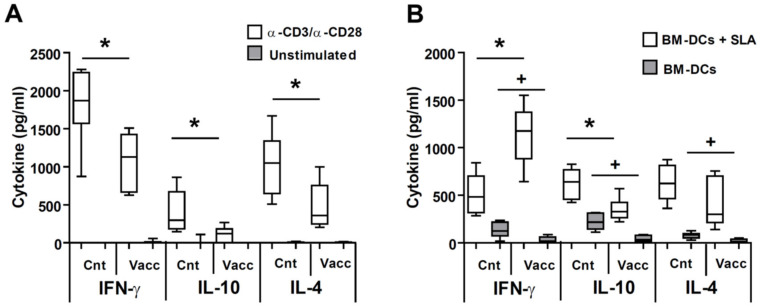
Analyses of the production of cytokines in the lymph node draining the site of *L. amazonensis* challenge. BALB/c mice were inoculated with PBS (control) or with 1 × 10^7^ *LiΔHSP70-II* attenuated promastigotes suspended in PBS (vaccinated) in the right footpad. At week 12 after vaccination control and vaccinated mice were challenged with 5 × 10^6^ stationary *L. amazonensis* promastigotes in the left footpad (*n* = 8). Animals were sacrificed at week 11 and the left popliteal lymph node cells were cultured in 96-well plates coated with anti-CD3 in complete medium supplemented with anti-CD28. As a control, similar cultures were performed in the absence of stimulating antibodies (**A**). Equivalent cultures were established and grown with BM-DCs loaded or not with SLA (**B**). The presence of IFN-γ, IL-10, and IL-4 was determined by ELISA in culture supernatants. Data are shown as whisker (min to max) plots. * (*p* < 0.05; BM-DCs + SLA) or ^+^ (*p* < 0.051; BM-DCs) shows the statistical differences between control and vaccinated groups (Mann–Whitney test). Results from one out of two similar experiments are represented.

Next, we analyzed the cytokine production in the left popliteal lymph node that drains the site of infection for the high dose model using anti-CD3 and anti-CD28 antibodies, as a global stimulus, or BM-DCs loaded or not with SLA as a parasite-specific stimulus. The production of the three cytokines studied (IFN-γ, IL-10, and IL-4) using T cell-stimulating antibodies was much higher in the animals of the control group than in vaccinated animals, correlating to the greater severity of the disease (Figure 4A). This increased immunological activity of the animals from the control group was also evidenced by co-cultivating the lymph node cells with BM-DCs alone, improving antigen presentation in an unspecific manner. Although the levels are moderate, the production of IFN-γ, IL-10 and IL-4 was significantly greater in the animals of the control group when compared with the vaccinated one (*p* = 0.0152, *p* = 0.0022, *p* = 0.0087, respectively) as shown in Figure 4B. The cytokine pattern changed radically when the response against the parasite proteins was analyzed. These data, shown in Figure 4B (BM-DCs + SLA), demonstrated a significant increase in the production of parasite-dependent IFN-γ (*p* = 0.0087) and a significant decrease in the production of IL-10 (*p* = 0.0087) in vaccinated animals relative to control ones. No significant differences were found in parasite-driven IL-4 production between control and vaccinated animals in the DLN (Figure 4B).

**Figure 5:**
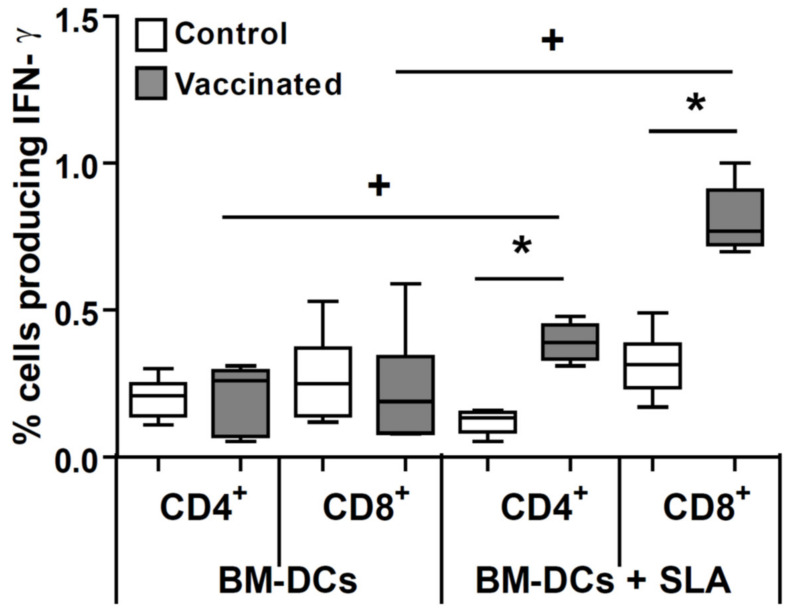
Involvement of T cells in the parasite-dependent production of cytokines. BALB/c mice were inoculated with PBS (control) or with 1 × 10^7^ *LiΔHSP70-II* attenuated promastigotes suspended in PBS (vaccinated) in the right footpad. At week 12 after vaccination control and vaccinated mice were challenged with 1 × 10^6^ stationary *L. amazonensis* promastigotes in the left footpad (*n* = 8). At week 11 after challenge the cells from the left popliteal lymph node were co-cultured with BM-DCs pulsed or not with SLA. Afterwards cells were processed for flow cytometry. The percentages of IFN-γ secreting cells in CD4^+^ or CD8^+^ gates are shown. Data are shown as whisker (min to max) plots. * (*p* < 0.05) shows the statistical differences between control and vaccinated groups; ^+^ (*p* < 0.05) shows the statistical differences between SLA-stimulated and unstimulated cells (Mann–Whitney test). Results from one out of two similar experiments are represented.

We next determined the presence of cells producing IFN-γ in the *L. amazonensis* infected popliteal lymph node in control and vaccinated mice. For this last group, we found a parasite-dependent production of this cytokine. Both CD4^+^ but specially CD8^+^ parasite-specific IFN-γ secreting T cells were detected in the *L. amazonensis* infected popliteal lymph node of the mice vaccinated with the attenuated line (additional file 2: Figure S2 and Figure 5).

## 4. Discussion

Until now, the only successful vaccination strategy against CL in humans has been leishmanization, which consists in the inoculation of *L. major* promastigotes into the skin. Upon self-healing of the CL lesion, individuals generate resistance against CL caused by the same or related species such as *L. tropica* [30]. This approach is difficult in the case of ACL due to the high pathogenicity of the species that cause it, including *L. amazonensis.* In addition, leishmanization experiments performed with low doses of parasites in the BALB/c mouse have shown that the inoculation of *L. amazonensis* does not generate homologous protection as is the case with *L. major* [31] or even with *L. infantum* [32]. A similar result was obtained when the C57BL/6 mouse model was employed. Contrary to what occurs after infection by *L. major*, the lesions are not eliminated spontaneously and the infection does not generate protection against a secondary challenge [33]. Interestingly, assays performed in the rhesus monkey model revealed that animals recovered from CL due *L. major* or *L. braziliensis* infection were protected against *L. amazonensis* infective challenge, opening up the possibility of carrying out cross-protection vaccination schedules. [34]. Since biosecurity concerns make problematic the use of infective forms, the use of attenuated cell lines offers the possibility of generate immunity without inducing the disease. This strategy, studied mainly for experimental models of VL [35] and CL caused by *L. major* and species for the *Viannia* subgenus like *L. braziliensis* [20] has not been extensively studied in the case of *L. amazonensis*.

Regarding the use of genetically modified attenuated parasite, the inoculation of the *L. major dhfr-ts*^-^ attenuated strain in murine models revealed that this strategy is feasible to partially protect in the short-term against an *L. amazonensis* challenge [23]. The results presented in this work reinforce this hypothesis demonstrating that vaccination with live attenuated vaccines was able to induce protection and, importantly, revealing that the achieved protection is maintained long-term. This is an important issue since single vaccination programs will help to maintain immunity in endemic regions of human leishmaniasis that usually coincide with disadvantaged regions in terms of health and economic resources. The choice of this attenuated *Leishmania* cell line for designing vaccines was based on our previous works demonstrating the strong short and long-term CD4^+^ and CD8^+^ T cell-mediated memory response found in vaccinated mice, that mediates protection against the heterologous challenge with *L. major* (BALB/c and C57BL/6 mice) [24,25] and the long-term capacity to reduce the severity of the chronic phase due to *L. infantum* challenge in BALB/c mice [26]. The ability to maintain long-term protection after curing the disease or after administration of an attenuated strain has been associated with concomitant immunity generated by the presence of a persistent population of parasites, formed by non-replicating parasites that eventually can resume replication, providing a source of antigens to keep parasite-specific T cells circulating [36,37]. Our previous data [25,26] and data shown in this work (Figure S1) demonstrate the continued presence of limited numbers of attenuated vaccine parasites in the lymph node draining the site of vaccination, while remaining absent in internal organs, as well as the site of infective challenge. This is an important issue in terms of biosecurity, since our results show that secondary infections do not cause the reactivation of the infection with the *LiΔHSP70-II* parasites or their dispersal throughout the whole organism. The low numbers of persistent functional parasites found in the popliteal draining the site of vaccination (below 100 parasites per lymph node; Figure S1 and reference [25]) are similar to those detected for the most evolved live attenuated vaccines based on *L. donovani* or *L. major* deficient in the centrin gene encoding a calcium binding cytoskeletal protein (*LdCen^-/-^* or *LmCen^-/-^*). The subsistence of these parasite lines was detected when administered either intradermically or s.c. in both hamster and mice models [38,39]. Regarding this point, we want to highlight that, in the *L. major*-C57BL/6 mice model, protection was correlated to the maintenance of IFN-γ-secreting effector T cells that were detected promptly in the site of infective challenge. In this sense, the presence of short-lived effector IFN-γ-secreting T cells (CD4^+^CD44^high^Ly6C^+^), previously associated with concomitant immunity [40], were associated to protection against a *L. major* challenge in *LmCen^-/-^* vaccinated mice [38]. Reinforcing the biosecurity of the *LiΔHSP70-II* vaccine, our previous data demonstrated that this line was unable to cause disease in hamsters, a model of infection that is highly susceptible when challenged with infective *L. infantum* promastigotes, or in SCID mice [24].

Regarding cross-protection, the fact that the attenuated vaccine studied in this work was generated from a *L. infantum* strain isolated from a dog affected by VL [41], does not represent a limitation regarding its use against different forms of leishmaniasis. As mentioned above, this strain was first characterized as cross-protective when studied in susceptible and resistant murine models of CL caused by *L. major* [24,25]. In addition, other attenuated strains based on viscerotropic species were characterized as protective against CL due to parasite species other than *L. amazonensis* [42,43]. There is also evidence of the protective capacity of subunit vaccines based on antigens of parasite species that cause VL, such as vaccines formulated with ribosomal proteins from *L. infantum* (LRP) [16], some domains of the *L. donovani* nucleoside hydrolase (NH) [44], or a recombinant multiantigenic-composed DNA vaccine (HisAK70) generated by different DNA regions encoding several proteins from *L. infantum* [18]. Finally, it was reported recently that inoculation of *L. infantum* parasites programmed for their destruction in the host (because of the expression of genes encoding proteins toxic for the parasite) protects BALB/c mice against a *L. amazonesis* challenge [22].

Concerning the protection achieved by the *LiΔHSP70-II* based vaccine our data show the existence of differences in the degree of protection depending on the number of parasites used for infective challenge. No cutaneous lesions were found in the mice receiving 5 × 10^4^ stationary-phase promastigotes, while some lesions were developed in mice receiving a higher inoculum size. This differential effect of the infectious dose on the evolution of the disease was not observed in other vaccine models such as C57BL/6 mice inoculated with a total protein-based vaccine LaAg [45]. On the other hand, the influence of the *L. amazonensis* inoculum size in the progression of the disease was previously reported for different murine models [6] and also influenced the studies regarding the effects of immunonutrition in the outcome of infection in BALB/c and C57BL/6 mice [46]. In the same direction, the protective effect of a vaccine based on the intranasal administration of extracellular serine proteases is inversely proportional to the infectivity of the inoculum [13]. It is difficult to make a comparative study of the efficacy of the *LiΔHSP70-II* vaccine in relation to other vaccines using attenuated parasites [23], vaccines based on the inoculation of inactivated *L. amazonensis* parasites [21] or subunit vaccines [14,16,18,44] because of the use of different inoculum sizes or *L. amazonensis* strains. However, our results demonstrate that a single administration of the *LiΔHSP70-II*-based vaccine is able to induce a similar, or even improved, protection in the long term compared to what other vaccines are able to provide in the short-term when a high dose model was employed.

It is a consolidated concept that protection against *L. amazonensis*, as with other species of the parasite, is related to the generation of an IFN-γ-mediated response against the parasite prompted by vaccination [47]. Our results demonstrated that protection induced by *LiΔHSP70-II* was also correlated to the induction of a Th1 systemic response against leishmanial antigens, evidenced by an increase of IFN-γ production in the spleen (Figure 3) and an increase in the titers of anti-SLA IgG2a circulating antibodies (Figure 2). These results are in line with other protective vaccines tested in the BALB/c model, and reinforce the assumption that increased parasite-dependent IFN-γ-mediated responses is a prerequisite for the development of a vaccine against *L. amazonensis* [8,13,17]. Furthermore, after infection, vaccinated mice showed lower titers of anti-*Leishmania* IgG1 antibodies than control unvaccinated mice. These data suggest that the protection observed was also related to the decrease in the systemic Th2 response against the parasite (Figure 2). Precisely, the animals challenged with the low dose, which presented a higher degree of protection, showed a significant decrease in the production of IL-4 in the spleen after stimulation with parasitic antigens compared to the controls (Figure 3). A decrease in the Th2 response against the parasite has been observed in some protective vaccines assayed in the *L. amazonensis* BALB/c model [13,14,17,18] but did not occur for other vaccines that also protected mice against disease development [8,9,48]. It can be concluded that the control of Th2 responses, although not an essential requirement to generate a protective vaccine against *L. amazonensis*, does appear to be associated with an increase in the Th1 response in some of the experimentally tested vaccines.

We show (Figure 4B) that, at the location of infection (popliteal DLN), control mice challenged with *L. amazonensis* secrete comparable amounts of parasite-specific IFN-γ, IL-4, and IL-10. These data are in accordance with others studies in which the immune response after challenge was studied in the DLN [48,49] or in the infected footpad [13]. This situation changes in the protected mice, where anti-parasite IFN-γ is the predominant secreted cytokine, as also occurred in mice vaccinated with other protective vaccines [18,48]. The percentage of both CD4^+^ and CD8^+^ T lymphocytes producing IFN-γ in the DLN is higher for the animals protected by vaccination with *LiΔHSP70-II* compared to their unvaccinated controls. This is a key observation, since it has been shown that protection against *L. amazonensis* in murine models correlated to the generation of both cell types secreting IFN-γ [44,50,51]. In this sense, protection induced by a DNA vaccine encoding for the parasite superoxide dismutase was associated to the induction of CD8^+^ T cells secreting IFN-γ [48]. Moreover, our results also evidence a decrease in the local production of IL-10 (Figure 4B), a cytokine related to the progression of the pathology [52,53,54], that was also decreased in the infected DLN of BALB/c mice protected against the disease by vaccination with total parasite extracts where cysteine proteases, known virulence factors, had been depleted [49]. The control of the local secretion of IL-10, would favor the ability of IFN-γ to activate the leishmanicidal capacities of *Leishmania*-infected macrophages and, therefore, contribute to the control of infection at the site of challenge.

## 5. Conclusions

It can be concluded that the administration of the *LiΔHSP70-II* vaccine is capable of controlling the progression of CL generated by the infection of *L. amazonensis* in BALB/c susceptible mice. Protection is evidenced by a control of the size of the skin lesions as well as a decrease in the parasite load at the site of infection and in its DLN with respect to control unvaccinated animals. The immune correlate to protection was the induction of parasite-specific IFN-γ responses, evidenced at the systemic and local levels, accompanied by the control of systemic Th2-mediated humoral responses and a reduction in IL-10 secretion at the local level. The data presented in this work highlight the protective capacities of the *LiΔHSP70-II* vaccine, since its immunization has been related to the induction of long-term protection against the infection of different species of parasites that cause CL (*L. major* and *L. amazonensis*) as well as infection with a viscerotropic species. In addition to the wide range of protection obtained by the use of this genetically altered cell line, the development of immunity in the absence of skin lesions typically found in leishmanized patients meets the ethical standards necessary for the design of a vaccine able to protect people for different forms of leishmaniasis.

## Data Availability

The data presented in this study are available in Figure 1, Figure 2, Figure 3, Figure 4 and Figure 5 and Supplementary Figures S1 and S2.

## Supplementary Materials

The following are available online at https://www.mdpi.com/2076-2607/9/2/363/s1, Figure S1: Presence of parasites in different organs and tissues. BALB/c mice were inoculated with PBS (control) or with 1 × 10^7^ *LiΔHSP70-II* attenuated promastigotes suspended in PBS (vaccinated) in the right footpad. At week 12 after vaccination animals were challenged with 5 × 10^6^ stationary *L. amazonensis* promastigotes in the left footpad. Parasite burden was individually determined by a limiting dilution assay in the liver, spleen and left or right popliteal lymph node at week 11 after challenge in the presence or the absence of the indicated antibiotics. Data are represented as whisker (min to max) plots. Figure S2: Analysis of lymph node T cell populations. Representative panels and gating strategy of experiments performed for obtaining data from Figure 5.
