# Supplementary figures and images for: Inoculation of the Leishmania infantum HSP70-II Null Mutant Induces Long-Term Protection against L. amazonensis Infection in BALB/c Mice
Source: Microorganisms. 2021 Feb 12;9(2):363. doi: 10.3390/microorganisms9020363 (PMC7918614; doi:10.3390/microorganisms9020363)

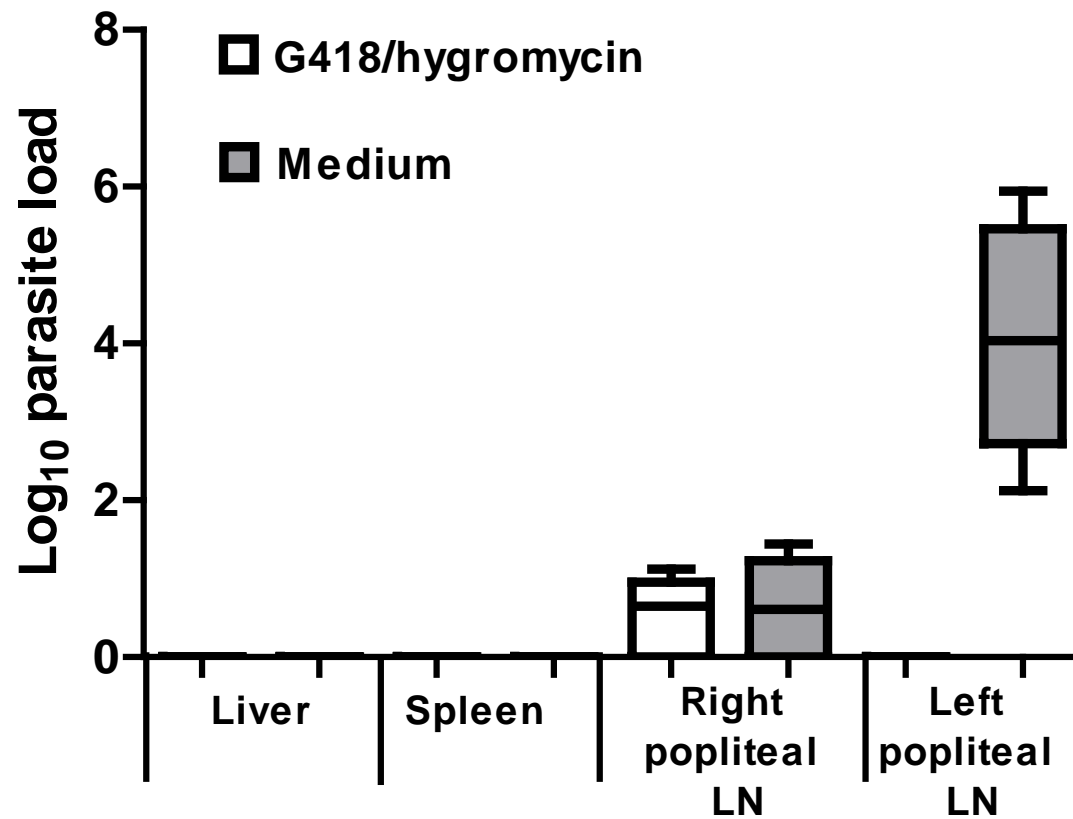

Supplement: Supplementary file 1 [file microorganisms-09-00363-s001.zip › SupplFigures/SF1.pdf]

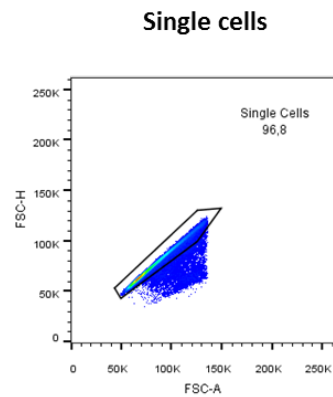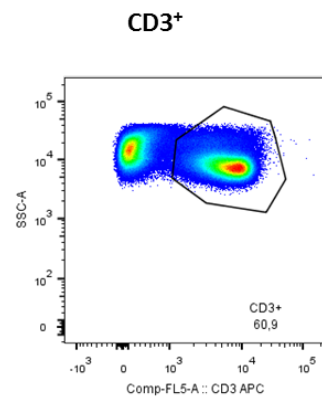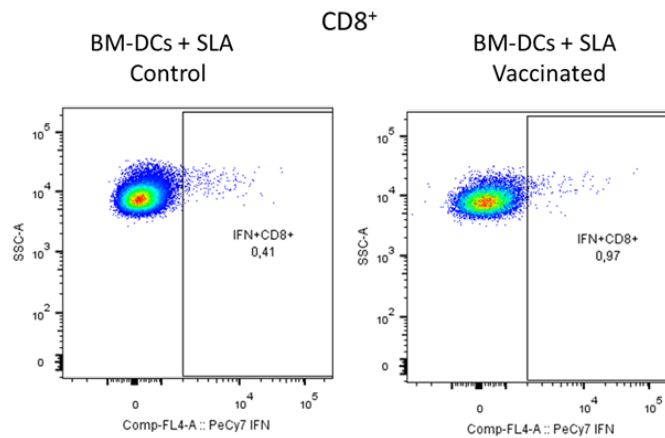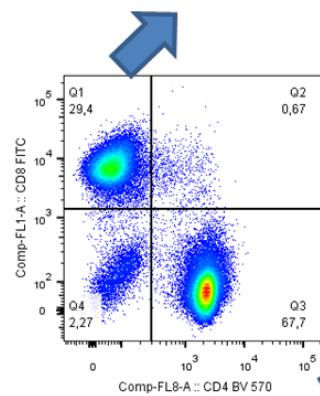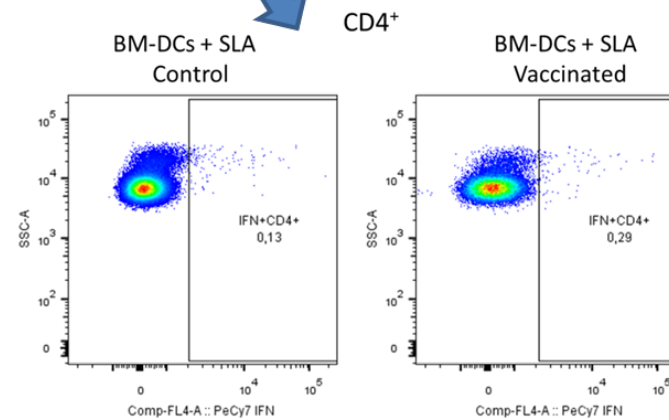

Supplement: Supplementary file 1 [file microorganisms-09-00363-s001.zip › SupplFigures/SF2.pdf]
